# Extensive Differences in Antifungal Immune Response in Two *Drosophila* Species Revealed by Comparative Transcriptome Analysis

**DOI:** 10.1155/2013/542139

**Published:** 2013-09-10

**Authors:** Yosuke Seto, Koichiro Tamura

**Affiliations:** ^1^Department of Biological Sciences, Tokyo Metropolitan University, 1-1 Minami-Osawa, Hachioji-shi, Tokyo 192-0397, Japan; ^2^Research Center for Genomics and Bioinformatics, Tokyo Metropolitan University, 1-1 Minami-Osawa, Hachioji-shi, Tokyo 192-0397, Japan

## Abstract

The innate immune system of *Drosophila* is activated by ingestion of microorganisms. *D. melanogaster* breeds on fruits fermented by *Saccharomyces cerevisiae*, whereas *D. virilis* breeds on slime flux and decaying bark of tree housing a variety of bacteria, yeasts, and molds. In this study, it is shown that *D. virilis* has a higher resistance to oral infection of a species of filamentous fungi belonging to the genus *Penicillium* compared to *D. melanogaster*. In response to the fungal infection, a transcriptome profile of immune-related genes was considerably different between *D. melanogaster* and *D. virilis*: the genes encoding antifungal peptides, Drosomycin and Metchnikowin, were highly expressed in *D. melanogaster* whereas, the genes encoding Diptericin and Defensin were highly expressed in *D. virilis*. On the other hand, the immune-induced molecule (IM) genes showed contrary expression patterns between the two species: they were induced by the fungal infection in *D. melanogaster* but tended to be suppressed in *D. virilis*. Our transcriptome analysis also showed newly predicted immune-related genes in *D. virilis*. These results suggest that the innate immune system has been extensively differentiated during the evolution of these *Drosophila* species.

## 1. Introduction

In natural environments, *Drosophila* species feed and breed on fermenting fruits, slime fluxes on decaying parts of tree, and so forth, where biochemical processes of bacteria and fungi are extremely active [1–3]. Therefore, *Drosophila* species are exposed to a huge number of microorganisms throughout their developmental stages. Feeding on decaying or fermented materials results in the ingestion of a wide variety of microorganisms in their digestive organs. Recent studies on larval immune response of *D. melanogaster* to oral infection of bacteria and fungi showed that the fat body mediated systemic immune response including antimicrobial peptide (AMP) production was triggered by infections of Gram-negative bacterial species such as *Pseudomonas entomophila* and *Erwinia carotovora carotovora* 15 (Ecc15) and of a dimorphic fungal species, *Candida albicans* [4–6]. 

AMPs are cationic small secretory peptides that exhibit a wide range of activities against bacteria, fungi, and/or viruses, playing an essential role in the innate immune system of *Drosophila* [7]. To date, seven AMP families, that is, Attacin, Cecropin, Defensin, Diptericin, Drosocin, Drosomycin, and Metchnikowin, have been identified in *Drosophila melanogaster* [7]. According to Sackton et al. (2007), it was indicated by their sequence analysis of the 12 *Drosophila* genomes that only the species belonging to the *melanogaster* species group of the subgenus *sophophora* had Drosomycin genes [8]. Drosomycin is known to be a major antifungal peptide [9–11]. This suggests that antifungal immune response varies among different *Drosophila* species and attacks from different bacteria and/or fungi might have produced different immune responses in *Drosophila*. Therefore, it is hypothesized that the differences in the environmental factors caused the difference in the immune system. 

**Figure 1:**
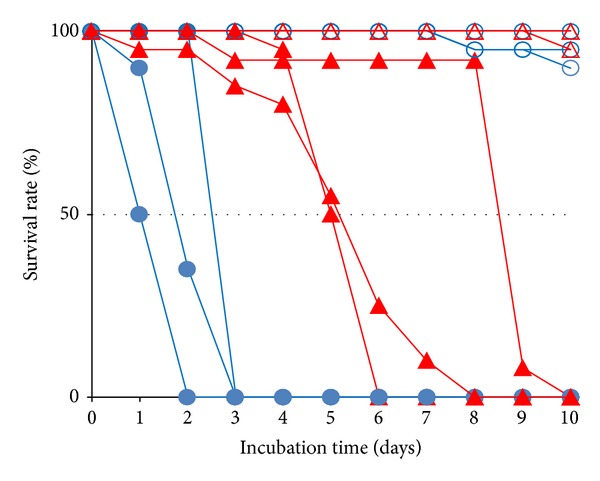
Survival curves of fungal-infected and naïve *D. virilis* and *D. melanogaster*. Twenty to twenty-five flies 1 day after eclosion were reared at 25°C on the culture medium covered by a *Penicillium* species (infected) or without fungus (naïve). The red lines with filled and open triangle data points indicate fungus-infected and naïve *D. virilis*, respectively, whereas the blue lines with filled and open circle data points indicate fungus-infected and naïve *D. melanogaster*, respectively.

For instance, *D. virilis* feeds and breeds on slime flux and decaying bark of trees, which are infected by various bacteria, yeasts and molds. Indeed, many yeasts, other than *Saccharomyces cerevisiae* and filamentous fungi, such as* Xanthophyllomyces dendrorhous*, *Cryptococcus* spp., and *Fusarium* spp., have been isolated from slime flux and decaying wood [14, 15], whereas *S. cerevisiae* solely ferments various fruits, which* D. melanogaster* thrives on [1–3]. From this difference in the microbial community in host materials of *D. virilis* and *D. melanogaster*, it is conceivable that *D. virilis* is exposed to a wider variety of fungi and therefore *D. virilis* has a higher resistance to fungi compared to *D. melanogaster*. To test this hypothesis, we examined the immune response of *D. virilis* and *D. melanogaster* to a fungus species belonging to the genus* Penicillium*. Since *Penicillium* species are commonly found in both slime flux and rotting fruits [16, 17], both *D. virilis* and *D. melanogaster* likely have high risk of *Penicillium* infection during all their developmental stages. To measure resistance of *D. virilis* and *D. melanogaster* to the fungal infection, adult flies of these species were reared on the culture medium that *Penicillium* fungi grew. The results showed that *D. virilis* adult flies survived more than two times longer than *D. melanogaster* flies (Figure 1), suggesting that *D. virilis* has a higher resistance to *Penicillium* infection. This higher antifungal activity without having Drosomycin motivated us to investigate the immune system of *D. virilis*.

In this study, to clarify the immune mechanism responsible for the higher antifungal resistance of *D. virilis*, larval immune response to the fungal infection between *D. virilis* and *D. melanogaster* were compared by means of comparative transcriptome analyses. Using a Roche 454 GS Junior sequencer, we examined the transcriptome of fat body and salivary gland of 3rd-inster larvae with and without infection of a *Penicillium* species. Genes showing different expression patterns in response to the fungal infection between *D. virilis *and *D. melanogaster* were extracted and compared. These genes included the genes encoding AMPs and “immune-induced molecule (IM).” Extensive differences were observed in the expression pattern of already known AMP and IM genes between *D. virilis* and *D. melanogaster*. Additionally, two potential AMP genes were newly identified from function-unidentified genes. Furthermore, three novel putative immune-related genes were identified: the products of them had a homology to an IM, Ras-like GTP-binding protein Rho1 involved in many signaling pathways and Ficolin-2 binding to a cell wall component of bacteria and fungi, respectively. 

## 2. Materials and Methods

### 2.1. Measurement of Antifungal Resistance

 Twenty to twenty-five adult flies 1 day after eclosion were reared at 25°C on a cornmeal-malt medium (50 g cornmeal, 50 g malt powder, 40 g dried brewer's yeast, 50 g sucrose, 5 mL propionic acid and 5 g agar in 1 liter water) with and without *Penicillium* fungi. The medium containing *Penicillium* fungi was prepared by inoculating a small amount of spores of a *Penicillium* species (identified by its nucleotide sequence of 18S RNA gene) onto the cornmeal-malt medium and incubated at 20°C for a week or more until the surface was completely covered by the growing fungi. After the flies were transferred onto the medium with or without fungi, the number of flies alive was counted every day. To measure the resistance to the infection of the *Penicillium* species, the 50% lethal time (LT50) was estimated by the generalized linear method implemented in R version 2.15.2 software [18]. These processes were independently replicated three times.

### 2.2. Induction of Gene Expression by Fungal Infection

A small amount of *Penicillium* spores were inoculated and cultured on a Sabouraud dextrose agar (SDA) medium (10 g peptone, 40 g dextrose, and 15 g agar in 1 liter water) at 20°C for several days until the fungi grew on to cover the surface of the medium. To prepare the fungus-infected larvae, twenty 3rd-instar larvae of *D. virilis* or *D. melanogaster* were reared on the fungus-covered SDA medium for 12 hours at 20°C. The induction of AMP genes is usually detected in three hours after the infection and continued at least 24 hours at 25°C [4, 6]. However, we reared the larvae at 20°C to postpone their pupation. The responses to the fungal infection was confirmed by the raised expression level of the Metchnikowin gene (known antifungal AMP gene) measured by RT-PCR and only the induction confirmed samples were used for the transcriptome sequencing described in the next section. As the control, the naïve larvae were prepared by rearing with the same condition on fungus-free SDA medium. 

### 2.3. Transcriptome Sequencing

 We analyzed transcriptome of larval fat body and salivary grand. This is because all AMPs were shown to be expressed in fat body and a major antifungal AMP, Drosomycin, was highly expressed in larval salivary gland in *D. melanogaster* [19]. Larval fat bodies and salivary glands dissected from twenty fungus-infected or naïve 3rd-instar larvae were pooled and the total RNA was extracted from these fat bodies and salivary glands by acid-guanidium phenol-chloroform (AGPC) method [20]. Then, mRNA was isolated by using Dynabeads mRNA purification kit (Invitrogen) according to the supplier's instruction. The complementary DNA (cDNA) library was constructed according to the Roche GS Junior cDNA rapid library preparation protocol with a modification to keep short molecules expected for AMP genes. The double-stranded cDNA was synthesized by using cDNA synthesis system (Roche Diagnostics) with random hexamer primers. The resultant cDNA was purified by using AMPure XP kit (Agencourt) and the end-polished cDNA fragments were ligated with the FAM-labeled RL adaptor included in Lib-L GS FLX Titanium Rapid Library Preparation kit (Roche Diagnostics). The adaptor-ligated cDNA was then purified by using Agencourt AMPure XP system and finally eluted in 50 *μ*L TE buffer. The cDNA solution was then concentrated by extracting with the equal volume of 2-butanol twice and subsequently with diethyl ether to remove the residual 2-butanol. Instead of the sizing procedure described in the standard protocol, we conducted 2% agarose-gel electrophoresis, excised the gel section containing 200 bp to 1 kb DNA fragments, and extracted the cDNA using High Pure PCR Clean-up kit (Roche diagnostics). The quality and quantity of the cDNA were evaluated by using QuantiFluor-P Handheld Fluorometer (Promega) and Agilent 2100 Bioanalyzer High Sensitivity DNA kit (Agilent Technologies). The pyrosequencing was conducted by using a 454 GS Junior sequencer after the emulsion PCR according to the manufacturer's instructions (Roche diagnostics).

### 2.4. Gene Prediction for Pyrosequencing Reads

**Figure 2:**
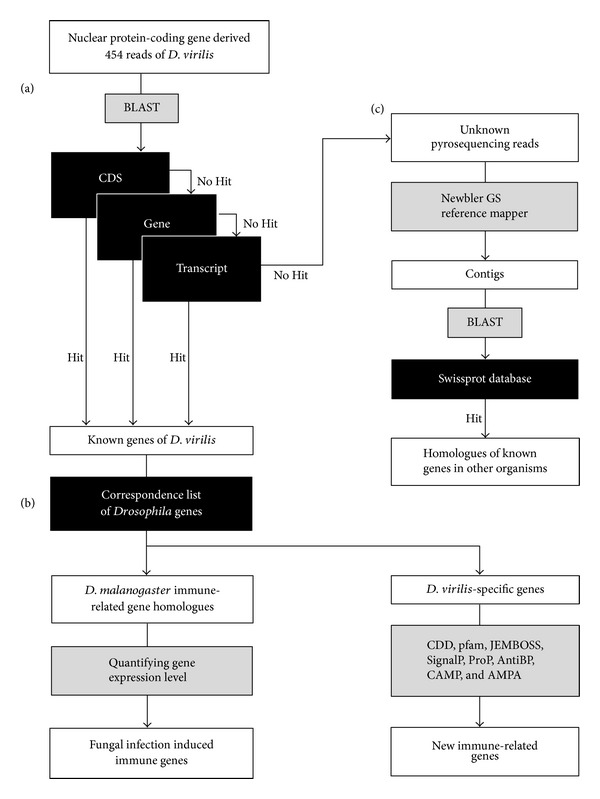
Workflow of data analyses for gene identification (a), gene expression (b), and prediction of immune-related gene (c). Input data in an open box is processed by program(s) in the grey box on the following arrow with or without a database in the black box leading to its outcome in the open box.

All the sequence reads obtained from a 454 GS Junior sequencer were filtered by the shotgun full processing of GS Run Processor application with the default setting. The filtered pyrosequencing reads of *D. melanogaster* and of *D. virilis* were queried to the complete mitochondrial genome sequence of *D. melanogaster* (FlyBase genome database release 5.46, ftp://ftp.flybase.net/genomes/) and that of *D. virilis* (NCBI; gi 190710421), respectively, by using the standalone BLAST 2.2.25+ software [21, 22] to remove the reads derived from mitochondrial genes. The reads that did not hit the mitochondrial genome sequence were then queried to *D. melanogaster* ribosomal RNA (rRNA) sequences (NCBI; gi 158246) to remove the reads from rRNA. To identify the gene, from which each read derived, each read was queried against the FlyBase* D. virilis* database release 1.2 or *D. melanogaster* database release 5.46 downloaded from FlyBase FTP site (ftp://ftp.flybase.net/genomes/), depending on which species it was derived from. Using the stand-alone BLAST 2.2.25+ software, we first queried against the CDS database and the reads that did not hit were subsequently queried against gene and transcript databases (Figure 2(a)). Finally, the reads that did not hit any target were used for further analyses to search for novel immune-related genes as explained later in Section 2.6.

For the genes identified in the *D. virilis* genome, most of them have different names from their orthologues in the *D. melanogaster* genome. In this study, however, we used the gene names of *D. melanogaster* for both species for the ease of comparison between species. The correspondence of gene ID between the two species was according to the 12 *Drosophila* genome analyses (ftp://ftp.flybase.net/genomes/12_species_analysis/clark_eisen/homology/) [23]. For genes that have multiple IDs corresponding to multiple copies in either or both species, one-to-one correspondence of homologue between the two species was determined by TBLASTN search with the translated protein sequence of *D. virilis* gene as the query against the *D. melanogaster* CDS database. Whether a gene is immune-related or not was determined by referring to the list of *Drosophila* immune-related genes [8]. 

The *D. virilis* genes of unknown function, which did not have homologue in the *D. melanogaster* genome, were further BLAST-searched for their homologues in other organisms' genomes (http://blast.ncbi.nlm.nih.gov/) [21]. In this homology search, only the genes, for which the number of reads was significantly different between fungus infected and naïve larvae, were used. For the genes that did not hit any homologue in any organism (*D. virilis*-specific genes), their functions were predicted by using domain and motif search programs available in NCBI Conserved Domain Database (http://www.ncbi.nlm.nih.gov/Structure/cdd/cdd.shtml) and Pfam (http://pfam.sanger.ac.uk/) (Figure 2(b)). When any conserved domain or motif was not predicted, the presence of signal peptide was predicted by using SignalP (v4.0) [24] and ProP (v1.0) [25] programs as a criterion to consider the possibility of antimicrobial peptide. For the candidates with putative signal peptide, the molecular weight, net charge, and structural features were computed by using JEMBOSS (v1.5) program [26]. Finally, from the amino acid sequence of putative mature peptide after removal of the putative signal peptide, the possibility of antimicrobial peptide was examined by AMP prediction web programs, AntiBP2 [27], CAMP [28], and AMPA [29]. 

### 2.5. Estimation of Gene Expression Level

To estimate the expression level of each gene, the total number of reads to hit the gene in the BLAST search was counted (Figure 2(b)). To calibrate the difference in transcript length among different genes, the number of reads counted was then standardized to be the number of reads per site per million reads (RPSM) as follows:

(1) RPSM=(number of reads/total number of readstranscript length )×1,000,000.  

We further normalized RPSM to take the difference in total gene expression level between the samples into account and computed trimmed Mean of *M* values (TMM) [30], using TCC package implemented in R version 2.15.2 software [18, 31]. For each gene, the TMM for the fungus infected larvae was compared to that for the control naïve larvae to quantify the extent of gene expression change in terms of the induction coefficient (IC) as follows:

(2) $$\begin{array}{c} \text{IC}=\frac{\text{TMM of the infected larvae}}{\text{TMM of the naïve larvae}}. \end{array}$$

To test the statistical significance of the induction, the difference in the number of actual reads was compared between the fungus infected and naïve larvae. In this test, RpL32 and GAPDH genes were used as endogenous control genes. Although actin was also a well-known endogenous control gene, actin was reported to play an important role in phagocytosis against fungi in *Drosophila* S2 cell [32] and that the expression of an actin gene (Act42A) of *D. melanogaster* 3rd-instar larvae was induced by *Saccharomyces cerevisiae* contained in the culture medium [33]. Indeed, the expression of *D. melanogaster* Act42A gene was not detected in the control naïve larvae but in the fungus infected larvae (the number of reads was 6 and TMM = 0.0619). Therefore, only RpL32 and GAPDH genes were used as the endogenous control genes in this study. Since the homogeneity of the numbers of reads for the two genes between the fungus infected and the naïve larvae was statistically supported (*P* = 0.14 in *D. virilis* and *P* = 0.51 in *D. melanogaster* by Fisher's exact test, Supplementary Table  1 available online at http://dx.doi.org/10.1155/2013/542139), the total number of reads derived from the two genes was used as the number of reads for the endogenous control genes. Finally, the difference in the number of reads between the fungus infected larvae and the naïve larvae was tested on the 2 × 2 contingency table with the numbers for the endogenous control genes by Pearson's chi-square test or Fisher's exact test dependent on whether the minimum number of reads was five or more or not.

### 2.6. Prediction of New Immune-Related Genes in *D. virilis*

The pyrosequencing reads which were derived from the fungus infected *D. virilis* but not mapped to any known gene were subject to predicting a new gene (Figure 2(c)). These pyrosequencing reads were mapped to the *D. virilis* genome sequence by Newbler GS reference mapper software (Roche Diagnostics) with the default parameter setting designated for CDS sequences to obtain continuous transcript sequences. Since the median length (192 bp) of the obtained contigs was similar to that (230 bp) of 3′-UTR of *D. melanogaster* [34], many contigs might not include protein coding region at all. Therefore, for each contig, the corresponding genome sequence plus 250 bp each of its upstream and downstream flanking regions were extracted to build a query sequence to search for new gene. All the query sequences obtained were subjected to BLASTX search against Swissprot protein database downloaded from the Uniprot web site (http://www.uniprot.org/downloads) with the condition of *e*-value ≤ 1*E*− 05. For the identified putative genes, the difference in the number of reads was statistically tested between the fungus infected and the naïve larvae in the same way as that for the known genes described above and if the number of reads was significantly different, then the gene ontology was analyzed by STRAP software (v1.1.0.0) [35].

## 3. Results

### 3.1. Difference in Antifungal Resistance between *D. virilis* and *D. melanogaster*

To compare antifungal resistance between *D. virilis* and *D. melanogaster*, adult flies of these species were reared on a culture medium harboring* Penicillium* fungi and their survival time was measured. The results showed that the *D. virilis* flies survived more than two times longer than the *D. melanogaster* flies did (Figure 1); the average 50% lethal times (LT50) of *D. virilis* and *D. melanogaster* flies were 6.04 days and 1.75 days, respectively, whereas their survival time on the normal culture medium without fungi was much longer (LT50 ≫ 10). This suggests that *D. virilis* has a higher resistance to the infection of the *Penicillium* species than *D. melanogaster* at the adult stage.

### 3.2. Transcriptome Analysis Summary

 Many AMP genes encode relatively short peptides less than 100 amino acids long. Therefore, to avoid the loss of sequences derived from such short transcripts, the 454 GS Junior sequencing was adjusted for cDNA library containing cDNA fragments longer than 200 bp long, whereas the standard sizing procedure selects DNA fragments of 600–900 bp long on average by removing those shorter than 350 bp long to be less than 10%. This resulted in 109,106 reads with the average length of 226 bp and 119,533 reads with the average length of 217 bp from the fungus infected and the naïve (uninfected) *D. virilis *larvae, respectively (Table 1). On the other hand, 110,578 reads with the average length of 242 bp and 91,947 reads with the average length of 219 bp were obtained from the fungus infected and the naïve (uninfected) *D. melanogaster *larvae, respectively (Table 1). 

After removing the reads derived from mitochondrial genes and rRNA genes, the total numbers of the remaining reads were 77,558 and 90,836 for the fungus infected and naïve *D. virilis* larvae, respectively, and 65,670 and 48,474 for the fungus infected and naïve *D. melanogaster* larvae, respectively. They were thought to be derived from mRNA transcribed from nuclear protein-coding genes. For 55,358 and 62,110 out of the 77,558 and 90,836 reads, respectively, we found BLAST hits for 5,155 and 4,709 genes, respectively, in *D. virilis*, whereas for 63,555 and 46,536 out of the 65,670 and 48,474 reads, respectively, we found BLAST hits for 4,735 and 4,275 genes, respectively, in *D. melanogaster*. It is noteworthy that the numbers of the remaining reads for *D. virilis* were 22,200 (fungus infected) and 28,726 (naïve), which were more than ten times as many as the corresponding 2,115 (fungus infected) and 1,938 (naïve) for *D. melanogaster* (Table 1).

### 3.3. Expression Pattern of Immune-Related Genes

 According to Sackton et al. (2007) [8], innate immune system is categorized into three functional classes: “recognition,” “signaling,” and “effector.” In the *D. virilis* transcriptome analysis, 128 immune-related genes were detected, in which 23, 68, and 37 were assigned to recognition, signaling, and effector classes, respectively (Table 2, Supplementary Table  2 and Supplementary Figure  1). In the case of the *D. melanogaster* transcriptome, 129 immune-related genes were detected, in which 28, 62, and 39 genes were assigned to recognition, signaling and effector classes, respectively (Table 3, Supplementary Table  3, Supplementary Figure  1). Among the immune-related genes, many of recognition and signaling class genes expressed in the fungus infected larvae were present in both *D. virilis* and *D. melanogaster* (Supplementary Figure  1). In the recognition class genes, PGRP-SA, PGRP-LC, PGRP-LE and GNBP3 involved in Toll and Imd pathways were expressed in both species. The expression of genes for nimrod and complement-like proteins called thioester-containing proteins (TEPs), which activate cellular immune response such as phagocytosis, were also detected in both species. Among the TEP genes, TEPII (IC = 5.359, *P* = 4.68*E* − 22) and TEPIV (IC = 2.515, *P* = 8.24*E* − 05) were significantly up-regulated in *D. melanogaster *(Table 3, Supplementary Table  3), whereas the expressions of their homologs in *D. virilis* were not induced by the fungal infection (Table 2, Supplementary Table  2). We also detected the genes for negative regulators of systematic immune response, such as PGRP-SC1a, PGRP-SC2, and PGRP-LB [36–39], as well as the genes for activators. Consistent with the expression of these recognition class genes, the expressions of signaling class genes, for example, Myd88, Rel, STAT92E, hep, and so forth, involved in Toll, Imd, JNK, and JAK/STAT pathways, were also detected in both species (see Tables 2 and 3 and Supplementary Tables  2 and 3 for details).

### 3.4. Between-Species Differences in the Expression Pattern of Effector Class Genes

 Since the effectors directly function against infected microbes, in this study, we focus on the response of the effector class genes to the *Penicillium* infection to elucidate the differences in the antifungal resistance between *D. melanogaster* and *D. virilis*. In contrast to the shared expression pattern between the species observed in the recognition and signaling class genes, substantial differences in the expression pattern were observed in the effector class genes.

**Figure 3:**
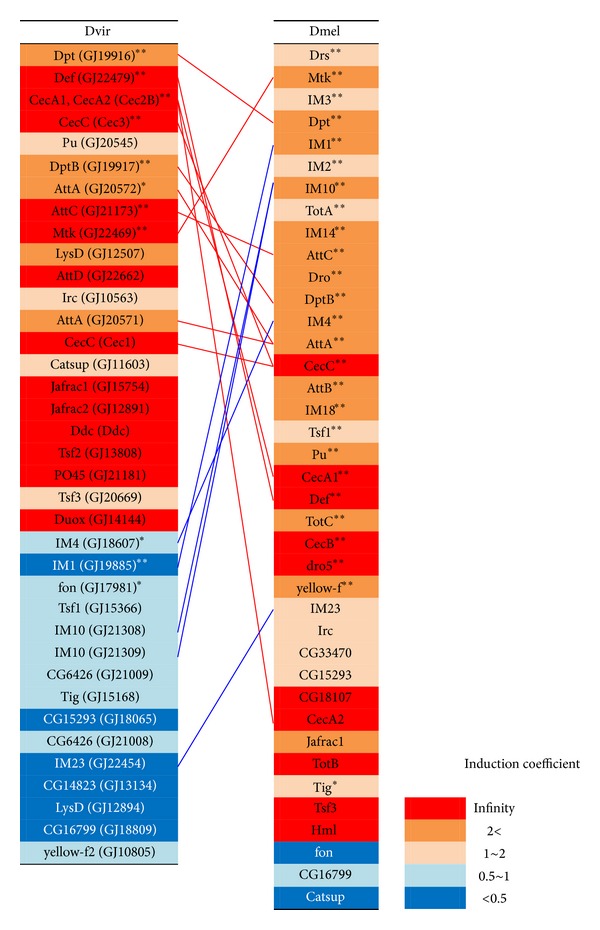
Summary of changes in gene expression level of the effector genes in the *Penicillium*-infected larvae. The effector class genes are piled in the order of the expression level in terms of trimmed mean of *M* values (TMM). Expressions of genes observed only in the *Penicillium*-infected larvae are displayed in red. Genes of the induction coefficient greater than 2.0, between 1.0, and 2.0, between 0.5 and 1.0 below 0.5 are displayed in dark orange, light orange, light blue, and dark blue, respectively. The AMP genes and the IM genes homologous between *D. virilis* and *D. melanogaster* are connected to each other by red lines and blue lines, respectively. For each *D. virilis* gene, the gene name of its homologue in *D. melanogaster* is described and the gene name of *D. virilis* is described in parenthesis. Asterisks indicate a statistically significant difference in the number of reads observed between the infected and naïve larvae (*P < 0.05; **P < 0.01).

 AMPs are known to be a major effector that has a critical role in the innate immune system of *Drosophila* [11]. In *D. melanogaster*, 20 AMP genes belonging to seven AMP gene families have been found, whereas 15 AMP genes belonging to five AMP gene families have been identified in *D. virilis* (Drosocin and Drosomycin in *D. melanogaster* are missing in *D. virilis*) [8]. In both *D. virilis* and *D. melanogaster*, many AMP genes (11 of 15 in *D. virilis* and 14 of 20 in *D. melanogaster*) were expressed in the fungus infected larvae (Tables 2 and 3, Supplementary Tables  2 and  3). In *D. virilis*, genes encoding Diptericin (GJ19916, TMM = 3.812), Defensin (GJ22479, TMM = 2.445), and Cecropin (Cec2B, TMM = 1.604 and Cec3, TMM = 1.475) showed high TMM values and Diptericin (GJ19916) was most highly expressed in the fungus infected larvae (Table 2). In contrast, the expression level of Metchnikowin, which was only the known antifungal peptide in *D. virilis*, was not so high (TMM = 0.660; Table 2). In contrast, Drosomycin (Drs) and Metchnikowin (Mtk), which were known as antifungal peptide genes, were most strongly expressed in the fungus infected *D. melanogaster* larvae (TMM = 23.817 and 23.719, resp.), followed by Diptericin (Dpt, TMM = 11.568), Attacin (AttC, TMM = 4.684), and Drosocin (Dro, TMM = 4.237) (Table 3). Among the Drosomycin gene family, only Dro5 responded to the fungal infection, suggesting that *D. melanogaster* uses the specific Drosomycin gene copy against the *Penicillium* species; However, the expression level of Dro5 was 100-fold lower than that of Drs (TMM = 0.276) (Table 3). These observations indicate substantial differences in the AMP usage between the species, that is, against the fungal infection, Diptericin, Defensin, and Cecropin were the three major AMPs in *D. virilis*, whereas Drosomycin and Metchnikowin were the two major AMPs in *D. melanogaster* (Figure 3). 

Among other effector class genes, the immune-induced molecule (IM) genes showed distinct expression pattern between the species. The IM genes are known as the genes induced by bacterial or fungal infection in *D. melanogaster*. However, their functions mostly have not been characterized. In this study, 10 IM genes were identified to be expressed in the fungus infected *D. melanogaster* larvae and five of them, IM1, IM4, IM10, IM14, and IM18, were significantly upregulated by 2-fold or more (Table 3 and Supplementary Table  3). For most of the *D. melanogaster* IMs, their expressions tended to be induced by the fungal infection. On the other hand, five IM genes, IM1 (GJ19885), IM4 (GJ18607), IM10 (GJ21308, GJ21309), and IM23 (GJ22454), were identified to be expressed in *D. virilis,* but their expression tended to be downregulated by the fungal infection (Table 2, Supplementary Table  2). Particularly, the expressions of IM1 (GJ19885), IM4 (GJ18607), and IM10 (GJ21308) were significantly reduced by the fungal infection by half or less (Table 2). These differences in the expression pattern may indicate that IMs play separate roles in the immune response to fungal infection in *D. melanogaster* and *D. virilis*. 

### 3.5. Novel AMP Genes in the Annotated *D. virilis* Genes

 Using the BLAST search against all the known *D. melanogaster* genes, we could not find the homologues for three *D. virilis* annotated genes significantly upregulated by the fungal infection. They were GJ10737 (IC = 2.503, *P* = 0.0037), GJ11722 (IC = 3.198, *P* = 0.032), and GJ18291 (IC = 3.909, *P* = 0.047). Additional queries to orthologue database (orthoDB: http://cegg.unige.ch/orthodb6) [40] and the nonredundant gene database in the NCBI BLAST web server failed to find any known gene, suggesting that they were *D. virilis*-specific genes. Although we further searched for annotated domains and motifs in the expected products of these genes using the domain and motif search programs on NCBI Conserved Domain Database and Pfam, no conserved domain or motif was predicted. However, using SignalP (v4.0) [24], ProP (v1.0) [25], and JEMBOSS (v1.5) [26] programs, the expected products of GJ10737 and GJ18291 were predicted to be secretory peptides having propeptide sequences and positively charged mature peptide (Table 4). These features are commonly found in AMPs. Indeed, AMP prediction web programs, CAMP [28] and AMPA [29], predicted them to be AMPs, although another program, AntiBP2 [27], did not (Table 4). These results suggested the possibility that *D. virilis* possesses unknown AMP genes functioning in its innate immune system.

### 3.6. Novel Immune-Related Genes in *D. virilis*

In our BLAST analysis described above, 22,200 and 28,726 pyrosequencing reads, respectively, from the fungal infected and naïve *D. virilis* larvae did not hit any known gene, whereas such reads were only 2,115 (infected) and 1,938 (naïve) in *D. melanogaster *(Table 1). We hypothesized that this is because there were many unidentified genes in *D. virilis*. To examine whether or not these reads were derived from unidentified immune-related genes, we assembled these reads by mapping each read onto the *D. virilis* genome sequence to make contigs. Then, we performed a BLASTX search against Swissprot protein database using each of these contigs as the query. 

**Figure 4:**
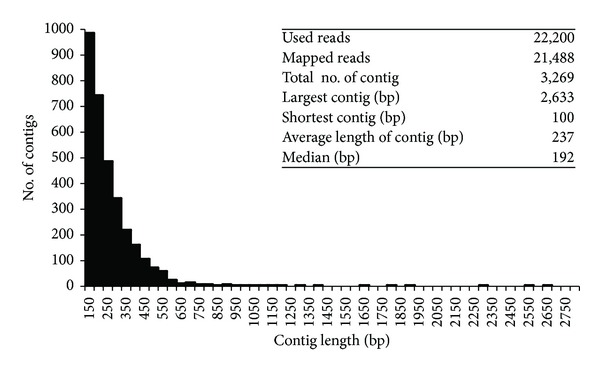
Distribution of sequence length (bp) of contigs constructed from the pyrosequencing reads of *D. virilis* that did not hit any annotated genes.

 Out of the 22,200 reads, 21,488 (about 97%) were mapped onto the *D. virilis* genome sequence to be assembled to 3,269 contigs of the average length 237 bp in total (Figure 4). This indicates that these reads were actually derived from transcripts of the *D. virilis* genome rather than possible contaminants and that there are unidentified transcription units potentially encoding polypeptide. Since most of the contigs were shorter than the median length of 3′-UTR of *D. melanogaster* genes, we extended each contig with 250 bp each of upstream and downstream genome sequences to make a query sequence subject to the BLAST search against Swissprot protein database. As a result, we identified 620 putative genes in the 3,269 contigs. Among them, 27 putative genes showed a statistically significant difference in the number of reads between the fungus infected and naïve larvae. Three out of the 27 putative genes, PG00034, PG01778, and PG02420, were assigned to potential immune-related genes for subsequent GO analysis (Supplementary Table  4). PG00034 was homologous to IM14 of *D. melanogaster.* Although the expression of IM14 was significantly up-regulated in *D. melanogaster* (Tables 3 and 5), the expression of PG00034 was significantly downregulated by the fungal infection in *D. virilis*. PG01778 was homologous to a Ras-like GTP-binding protein, Rho1, of *D. melanogaster*. This gene is known to play a role in regulating actin genes involved in phagocytosis [41–44]. The expression was observed only in the infected larvae in *D. virilis* and induced by the fungal infection (IC = 2.020) in the *D. melanogaster* larvae, indicating that this gene was up-regulated by the fungal infection in both species. PG02420 was homologous to Ficolin-2 that binds to the cell wall component of bacteria and fungi [45, 46], and the expression of PG02420 was significantly down-regulated in the infected *D. virilis* (IC = 0.208) (Table 5).

## 4. Discussion

In this study, we first clarified that the antifungal resistance against *Penicillium* fungal infection is higher in *D. virilis* than in *D. melanogaster.* In general, adult flies of most *Drosophila* species are attracted to, feed, and breed upon a variety of fermenting substances such as fallen fruit and flowers, slime fluxes of forest trees, decaying bark of trees, and mushrooms [1]. However, there are interspecies variations of the fermenting substances utilized by *Drosophila* species for feeding and breeding. For instance, *D. virilis* is known to feed on slime flux and decaying bark of tree harboring many yeasts and filamentous fungi, such as *Xanthophyllomyces dendrorhous*, *Cryptococcus* spp., and *Fusarium* spp. [14, 15], whereas *D. melanogaster* feeds on fermented fruits, which mainly harbor Baker's yeast, *Saccharomyces cerevisiae* [1–3]. The *Penicillium* species is ubiquitously and abundantly found in natural environment, where *Drosophila* species live, and grows on both decaying woods and fruits [16, 17]. Therefore, both *D. virilis* and *D. melanogaster* are likely to be infected by them in nature during their life time. According to the theory of evolutionary adaptation, the higher antifungal resistance of *D. virilis* observed in this study (Figure 1) is expected to reflect the result of higher risk of the infection in their living environments over the evolutionary time compared to *D. melanogaster*. This raises the question of the immune mechanism attributed to the higher antifungal resistance of *D. virilis*, and it is thought to be a key factor for understanding the adaptive evolution of *D. virilis* to its habitat in moldy environment. To answer this question, we compared the immune responses to the fungal infection between *D. virilis* and *D. melanogaster* by analyzing their transcriptome extracted from larval salivary gland and fat body. Although the antifungal resistance was compared at the adult stage, we focused on the transcriptome at the larval stage. Since the larvae live and feed on fermented substances in their habitat environment and cannot escape from the surrounding microbes as the adults fly away, the larvae are consistently infected by microbes. Therefore, we assume that the resistance at the larval stage is more important for their adaptation to the environment. Unfortunately, it was difficult to measure the antifungal resistance at the larval stage since the larvae became pupae within several days and some larvae avoided immediate infection of fungi by digging the medium deeply. Accordingly, our interpretation in the following is on the basis of the assumption that the resistance at the adult stage correlates with the resistance at the larval stage.

Our comparative transcriptome analysis revealed that the genes involved in all major signaling pathways for immune response, that is, Toll, Imd, JAK/STAT, and JNK, were triggered by the infection of the *Penicillium* species in both *D. virilis* and *D. melanogaster* (Tables 2 and 3, Supplementary Tables  2 and 3). These pathways regulate humoral and cellular immune responses, such as AMP production and phagocytosis [7, 47, 48]. Among the signaling pathways, the Toll pathway plays an essential role against fungal infection in *D. melanogaster* [10, 49]. The Toll pathway regulates expressions of two antifungal peptides, Drosomycin and Metchnikowin [50]. Consistent with this fact, the expression levels of Drosomycin and Metchnikowin genes were highest in the fungus infected *D. melanogaster* larvae (Table 3). The response of these AMP genes to the infection of an entomopathogenic fungus, *Beauveria bassiana*, was highest in adult *D. melanogaster* as well [51, 52]. Interestingly, seven genes encoding Drosomycin have been found in *D. melanogaster* genome (Drs, Drsl, Dro2, Dro3, Dro4, Dro5, and Dro6) [8]. Nevertheless, we found that only Drs and Dro5 were induced by the fungal infection in the *D. melanogaster* larvae (Table 3). This specificity of the expression pattern was consistent with the result of the microarray analysis by De Gregorio et al. (2001) [51], suggesting that the specific genes, Drs and Dro5, are used against the fungal infection at both larval and adult stages. In contrast, any Drosomycin gene is absent in the *D. virilis* genome and the expression of the Metchnikowin gene (Mtk) was not high (TMM = 0.660) compared to that of other AMP genes in the fungus infected *D. virilis* larvae (Table 2, Supplementary Table  2, Figure 3). This result was rather unexpected since Metchnikowin was the only known antifungal peptide in *D. virilis*, suggesting that Metchnikowin of *D. virilis* does not compensate for the lack of Drosomycin. Since the comparison of *D. melanogaster* and *D. virilis* genomes revealed that Mtk is present as a single copy gene in both species [8], it is implausible that *D. virilis* has an additional copy of Mtk responsible for the observed higher antifungal resistance.

**Figure 5:**
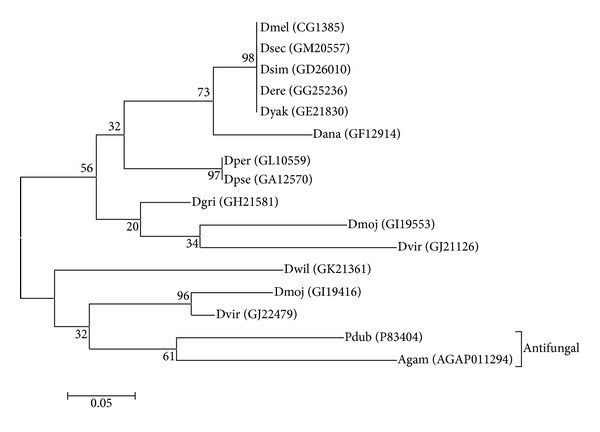
Neighbor-joining phylogenetic tree of *Drosophila* Defensin genes with antifungal Defensin genes of sand fly (*Phlebotomus duboscqi*) and mosquito (*Anopheles gambiae*). Amino acid sequences of the mature peptide were aligned by CLUSTAL W [12] and the phylogenetic tree was reconstructed with the Poisson model by MEGA5 [13]. For each Defensin gene, abbreviated four-letter species code (Dmel: *Drosophila melanogaster*, Dsec: *D. sechellia*, Dsim: *D. simulans*, Dere: *D. erecta*, Dyak: *D. yakuba*, Dana: *D. ananassae*, Dper: *D. persimilis*, Dpse: *D. pseudoobscura*, Dgri: *D. grimshawi*, Dmoj: *D. mojavensis*, Dvir: *D. virilis*, Dwil: *D. willistoni*, Pdub: *Phlebotomus duboscqi* and Agam: *Anopheles gambiae*) with Gene ID or Uniprot ID in parenthesis is shown as an operational taxonomic unit. The Defensins genes of *D. melanogaster* and *D. virilis* were indicated by bold face. The number along each branch is the bootstrap value computed by 1,000 bootstrap replicates.

On the other hand, the genes encoding Diptericin (GJ19916), Defensin (GJ22479), and Cecropin (Cec2B and Cec3) were highly expressed (TMM = 3.812, TMM = 2.445, TMM = 1.604 and TMM = 1.475, resp.) in the fungus infected *D. virilis* larvae compared to other AMP genes (Table 2), suggesting a substantial difference in the AMP usage in response to the fungal infection between the two species and a possibility that Diptericin, Defensin, and Cecropin have an antifungal function in *D. virilis*. The antifungal activity of Diptericin and Defensin against an ascomycete fungus, *Fusarium oxysporum*, has been reported, although they are not effective against other fungi (*Neurospora crassa*, *Beauveria bassiana,* and *Aspergillus fumigatus*) in *D. melanogaster* [11]. Comparing the Diptericin protein sequence of *D. virilis* to its orthologue in *D. melanogaster,* we found substantial amino acid differences (50–70%). This may indicate the possibility that Diptericin of *D. virilis* has a different activity spectrum against fungi from that of *D. melanogaster*, although the main activity of the latter is not antifungal but antibacterial [53]. In contrast, amino acid sequences of mature peptide of Cec2B and Cec3 of *D. virilis* are almost identical (92.5–100%) to those of Cecropin of *D. melanogaster*, and the few amino acid substitutions observed are all conservative to maintain physicochemical properties of the peptide. Therefore, it is likely that the functions of Cecropin are conserved in the two species. A notable difference was observed in the Defensin gene. Defensin is known to be an AMP of main specificity to Gram-positive bacteria in *D. melanogaster* [54]. However, the *Drosophila* Defensin is classified into Defensin_2 superfamily (Pfam: PF01097), which has antifungal activity in mosquito (*Anopheles gambiae*) and sand fly (*Phlebotomus duboscqi*) [55, 56]. *D. virilis *has two Defensin genes (GJ21126 and GJ22479). The mature peptide sequence of GJ21126 is closely related to the *D. melanogaster* Defensin gene as expected from their phylogenetic relationship of species, whereas the mature peptide sequence of GJ22479 is more similar to those of *Anopheles gambiae* (AgaDef) and *Phlebotomus duboscqi* (PduDef), which have antifungal activity (Figure 5). In our transcriptome analysis, we detected the expression of GJ22479 but not GJ21126 in response to the *Penicillium* infection. A possible speculation based on these observations is that Defensin functions differently as an antifungal peptide in *D. virilis* from that in *D. melanogaster*. Since the expression of these three AMPs is under the regulation of the Imd pathway rather than the Toll pathway [50, 57], this result suggests that the Imd pathway plays an important role in the response to the fungal infection in *D. virilis*, in contrast to the fact that the Toll pathway is more important to regulate the Drosomycin genes as the antifungal response in *D. melanogaster*. Alternatively, the Diptericin, Defensin, and Cecropin genes may be under the Toll pathway regulation in *D. virilis*. To examine this possibility, we analyzed the upstream region of these genes to see differences in DIF (Toll pathway) and Relish (Imd pathway) binding sites [58] between *D. virilis* and *D. melanogaster*. However, there was no clear difference in the number, position, and direction of these binding sites, suggesting that the alternative possibility is not likely.

A striking difference in the expression pattern was observed in the immune-induced molecule (IM) genes. The IM genes of *D. melanogaster* showed a similar expression pattern to that observed in the previous study conducted by De Gregorio et al. (2001) [51]. In this study, ten IM genes were expressed in the fungus infected *D. melanogaster* larvae and five of them, IM1, IM4, IM10, IM14, and IM18, were significantly up-regulated by 2-fold or more and down-regulated gene was not observed (Table 3, Supplementary Table  3). Similar inductions of IM genes were observed in adult flies by the infection of *B. bassiana* [51]. This suggests that the IM genes play a similar role in antifungal immunity in larvae and adults of *D. melanogaster* and against *Penicillium* and *Beauvaria* fungi, although the function of the IM genes has not been characterized. However, the IM genes showed contrary expression pattern in *D. virilis*: the expressions of five IM genes, IM1 (GJ19885), IM4 (GJ18607), IM10 (GJ21308, GJ21309), and IM23 (GJ22454), detected in *D. virilis,* were rather down-regulated by the fungal infection (Figure 3). Indeed, three of them, IM1 (GJ19885), IM4 (GJ18607), and IM10 (GJ21308), showed statistically significant reductions (Table 2, Supplementary Table  2). This result suggests differences in the functions of IMs between *D. virilis* and *D. melanogaster*. In other words, the definition of immune-induced molecule (IM) holds true in *D. melanogaster* but not necessarily so in other *Drosophila* species. It can be speculated that *D. virilis* may have other immune-related genes that have the functions of IMs in* D. melanogaster*. Based on the comparative transcriptome analysis using bacterial-infected *D. melanogaster* and *D. virilis* flies, Sackton and Clark (2009) suggested that new components were recruited into the immune system of *D. virilis* [34]. Therefore, our results as well as their observation motivated us to search for novel immune-related genes in *D. virilis*. 

In our transcriptome analysis, we found that three *D. virilis*-specific genes were induced by the fungal infection and two of them, GJ10737, and GJ18291, were predicted to encode novel AMPs (Table 4). This suggests that *D. virilis* has acquired lineage-specific AMPs against fungal infection through its evolution. Since no orthologous sequences of these genes were found in other *Drosophila* genomes either, these genes seemed to be recruited to the *D. virilis* genome de novo. In addition to the fraction of these genes of unknown function, we also predicted new *D. virilis* genes from the pyrosequencing reads that did not show any BLAST hit. 

In our BLAST analyses of the pyrosequencing reads, approximately 30% of the reads from *D. virilis* did not hit any gene, whereas only 3-4% of the reads from *D. melanogaster* fell in the same situation (Table 1). This may suggest the possibility that many genes in the *D. virilis* genome have not been identified yet. Actually, we found 620 putative genes in 3,469 contigs and three of them, PG00034, PG01778 and PG02420, were predicted to be immune-related genes with expression level significantly changed by the fungal infection. PG00034 is homologous to IM14 and PG01778 is homologous to a Ras-like GTP-binding protein, Rho1, which regulates actin cytoskeletal organization [41, 42] and is involved in phagocytosis [43, 44] in *D. melanogaster* (Table 5). PG02420 is homologous to Ficolin-2 of *Bos taurus*. Ficolin binds to a cell wall component of bacteria and fungi and is involved in phagocytosis [45, 46]. Although the expression of the IM14 gene was significantly up-regulated by the fungal infection in the *D. melanogaster *larvae, the expression of PG00034 was significantly down-regulated as in the case of other homologues of IM genes in the *D. virilis* larvae. Similarly, the expression of PG02420 was significantly down-regulated in the infected *D. virilis* larvae. On the other hand, the expression of PG01778 was significantly up-regulated by the fungal infection in *D. virilis*. For the remaining 2,649 contigs, we could not find any homologue in Swissprot protein database. This seems partly to be because many of them are too short to find a homology to a known gene, domain, or motif in the homology search (Figure 4). Further experimental determination of their full length sequence is necessary for a better prediction of novel protein-coding genes. Therefore, there is a possibility that some of these putative genes constitute novel components in the immune systems of *D. virilis* and contribute to the higher resistance against the fungal infection. 

Our comparative transcriptome analysis revealed extensive differences in the immune response to the infection of *Penicillium* species between *D. virilis* and *D. melanogaster* at the transcriptome level. These results provide an important insight into the different role of immune system between ecologically diverged species. It is quite natural to consider that the observed differences resulted from evolutionary adaptation to their different habitats. This presumption should be further experimentally examined by the investigation of antimicrobial activities of AMPs, for example, Diptericin and Defensin, to identify the component responsible for the higher antifungal resistance of *D. virilis*.

## 5. Conclusion

In general, *Drosophila* species feed and breed on fermenting fruits, slime fluxes on decaying parts of tree, and so on, in which a variety, of microbes are extremely active [1–3]. Therefore, antimicrobes immune system is an essential trait for *Drosophila* species to survive. The evolution of the immune system is likely responsible for the diversity of *Drosophila* species adapting to a variety of microbial environments. In this study, a substantial difference in antifungal activity against a *Penicillium* species between two *Drosophila* species, *D. virilis* and *D. melanogaster,* living in different environments, was demonstrated.

Our comparative transcriptome analysis showed extensive differences in the expression pattern of immune-related genes, that is, antimicrobial peptide (AMP) and the immune-induced molecule (IM) genes, in response to the *Penicillium* infection between *D. virilis* and *D. melanogaster*. Furthermore, we predicted novel immune-related genes responding to the fungal infection in *D. virilis*. These results indicate that the innate immune system has been substantially differentiated during the evolution of these *Drosophila* species. The extensive differences in the immune system may have been evolved as an adaptive response to microbial environments, which remains open to further investigations. 

## Supplementary Material

Supplementary Table 1: Number of reads, trimmed mean of M value (TMM) and induction coefficient (IC) for endogenous control genes in *D. virilis* and *D. melanogaster*.
Supplementary Table 2: Number of reads, trimmed mean of M value (TMM) and induction coefficient (IC) for recognition, signaling and effector class immune-related genes observed in *D. virilis*.
Supplementary Table 3: Number of reads, trimmed mean of M value (TMM) and induction coefficient (IC) for recognition, signaling and effector class immune-related genes observed in *D. melanogaster*.
Supplementary Table 4: Number of reads, induction coefficient (IC) and predicted function of the putative genes (PG) in *D. virilis*.
Supplementary Figure 1: Venn diagrams that represent the numbers of expressed immune-related genes for recognition (a), signaling (b) and effectors (c) observed in the Penicillium-infected *D. virilis* (Dvir) and *D. melanogaster* (Dmel) larvae. The numbers in parentheses indicate the numbers of duplicated genes in *D. virilis*.

## Figures and Tables

**Table 1:** Summary of statistics of 454 GS Junior sequencing and BLAST analysis.

	*D. virilis *		*D. melanogaster *	
	Infected	Naïve	Infected	Naïve
Total no. of reads	109,106	119,533	110,578	91,947
Maximum length (bp)	715	667	710	580
Minimum length (bp)	40	40	40	40
Average length (bp)	226	217	242	219

No. of mtDNA-derived reads	5,557	6,197	5,998	7,483
No. of rDNA-derived reads	25,991	22,500	38,910	35,990
No. of other reads	77,558	90,836	65,670	48,474
No. of BLAST hits (No. of genes)	55,358 (5,155)	62,110 (4,709)	63,555 (4,735)	46,536 (4,275)
No. of unidentified reads	22,200	28,726	2,115	1,938

**Table 2:** Number of reads, trimmed mean of *M* value (TMM), and induction coefficient (IC) for recognition, signaling, and effector class immune genes showing significant changes in expression level by fungal infection in *D.  virilis*.

*D. virilis* gene	*D. melanogaster* homologue	Infected		Naïve		IC	Functional class	Notes
		No. of reads	TMM	No. of reads	TMM			
GJ20666	CG13422	6	0.153	0	0	Infinity	Recognition	Beta-glucan binding domain
GJ12160	PGRP-SB1	11	0.235	2	0.040	5.864	Recognition	PGRP domain
GJ18074	nimB3	2	0.067	12	0.376	0.178	Recognition	Nimrod-related

GJ12373	msn	9	0.024	1	0.002	9.595	Signaling	Kinase
GJ20603	Pvr	15	0.038	2	0.005	7.996	Signaling	Receptor
GJ19441	SPE	3	0.033	15	0.155	0.213	Signaling	Protease

GJ22479	Def	53	2.445	0	0	Infinity	Effector	Antimicrobial peptide
GJ21173	AttC	47	0.818	0	0	Infinity	Effector	Antimicrobial peptide
Cec2B	CecA1/CecA2	25	1.604	0	0	Infinity	Effector	Antimicrobial peptide
Cec3	CecC	23	1.475	0	0	Infinity	Effector	Antimicrobial peptide
GJ22469	Mtk	9	0.660	0	0	Infinity	Effector	Antimicrobial peptide
GJ19916	Dpt	104	3.812	4	0.138	27.720	Effector	Antimicrobial peptide
GJ19917	DptB	39	1.120	3	0.081	13.860	Effector	Antimicrobial peptide
GJ20572	AttA	49	0.856	24	0.393	2.177	Effector	Antimicrobial peptide
GJ17981	fon	217	1.641	370	2.624	0.625	Effector	Coagulation
GJ18607	IM4	79	7.542	151	13.521	0.558	Effector	IM
GJ21308	IM10	23	0.350	51	0.727	0.481	Effector	IM
GJ19885	IM1	37	3.302	123	10.296	0.321	Effector	IM

Genes are sorted in order of induction coefficient at each functional class.

**Table 3:** Number of reads, trimmed mean of *M* value (TMM), and induction coefficient (IC) for recognition, signaling, and effector class immune genes showing significant changes in expression level by fungal infection in *D.  melanogaster*.

*D. melanogaster* gene	Infected		Naïve		IC	Functional class	Notes
	No. of reads	TMM	No. of reads	TMM			
PGRP-SB1	29	0.779	0	0	Infinity	Recognition	PGRP domain
PGRP-SC1b	11	0.288	0	0	Infinity	Recognition	Amidase degradation
PGRP-SB2	9	0.225	0	0	Infinity	Recognition	PGRP domain
Mcr	4	0.011	0	0	Infinity	Recognition	Tep
PGRP-SC2	20	0.603	3	0.102	5.891	Recognition	Amidase degradation
TepII	188	0.708	31	0.132	5.359	Recognition	Tep
nimC2	43	0.310	9	0.073	4.222	Recognition	Nimrod-related
GNBP3	15	0.164	4	0.049	3.313	Recognition	Beta-glucan binding domain
CG13422	17	0.569	5	0.189	3.004	Recognition	Beta-glucan binding domain
TepIV	37	0.131	13	0.052	2.515	Recognition	Tep
PGRP-SD	27	0.626	13	0.341	1.835	Recognition	PGRP domain

Rel	14	0.067	0	0	Infinity	Signaling	Transcription factor
aop	6	0.026	0	0	Infinity	Signaling	Transcription factor
brm	5	0.016	0	0	Infinity	Signaling	Transcription factor
Myd88	4	0.019	0	0	Infinity	Signaling	—
CG6361	15	0.185	1	0.014	13.254	Signaling	Protease
cact	11	0.081	1	0.008	9.720	Signaling	—
dom	8	0.085	1	0.012	7.069	Signaling	Transcription factor
Stat92E	11	0.050	3	0.016	3.240	Signaling	Transcription factor
srp	18	0.080	5	0.025	3.181	Signaling	Transcription factor
phl	32	0.135	9	0.043	3.142	Signaling	—
mask	10	0.012	3	0.004	2.945	Signaling	—
spirit	22	0.231	7	0.083	2.777	Signaling	Protease

CecC	35	1.521	0	0	Infinity	Effector	Antimicrobial peptide
CecA1	14	0.663	0	0	Infinity	Effector	Antimicrobial peptide
Def	11	0.461	0	0	Infinity	Effector	Antimicrobial peptide
CecB	7	0.288	0	0	Infinity	Effector	Antimicrobial peptide
dro5	6	0.276	0	0	Infinity	Effector	Antimicrobial peptide
AttC	252	4.684	2	0.042	111.333	Effector	Antimicrobial peptide
Dpt	343	11.568	24	0.916	12.628	Effector	Antimicrobial peptide
DptB	80	2.974	6	0.252	11.781	Effector	Antimicrobial peptide
Pu	79	0.687	7	0.069	9.972	Effector	Melanin synthesis cascade
TotC	10	0.311	1	0.035	8.836	Effector	Tot
IM18	62	1.403	8	0.205	6.848	Effector	IM
Mtk	380	23.719	52	3.673	6.457	Effector	Antimicrobial peptide
Dro	192	4.237	27	0.674	6.283	Effector	Antimicrobial peptide
yellow-f	23	0.277	6	0.082	3.387	Effector	Melanin synthesis cascade
IM14	68	5.101	19	1.613	3.162	Effector	IM
AttA	96	2.113	27	0.673	3.142	Effector	Antimicrobial peptide
IM4	56	2.194	16	0.709	3.093	Effector	IM
IM10	355	6.147	116	2.273	2.704	Effector	IM
IM1	247	11.541	82	4.336	2.662	Effector	IM
AttB	74	1.428	27	0.590	2.422	Effector	Antimicrobial peptide
IM2	139	6.250	62	3.155	1.981	Effector	IM
Tsf1	145	1.209	68	0.642	1.884	Effector	Iron binding
TotA	182	5.213	98	3.177	1.641	Effector	Tot
Drs	551	23.817	299	14.627	1.628	Effector	Antimicrobial peptide
Tig	22	0.053	12	0.033	1.620	Effector	Coagulation
IM3	330	18.401	188	11.864	1.551	Effector	IM

Genes are sorted in order of induction coefficient at each functional class.

**Table 4:** Trimmed mean of *M* value (TMM), induction coefficient (IC), number of amino acids of mature peptide, molecular weight, net charge and protein structural feature for putative antimicrobial peptide genes in *D. virilis* predicted by AMP prediction programs.

*D. virilis* gene	TMM	IC	Mature peptide size (aa)	Molecular weight (kDa)	Net charge	Structural features	AMP prediction		
							AntiBP2	CAMP	AMPA
GJ10737	1.368	2.503	35	4.07	12	Arg + Val rich (51%)	−	+	+
GJ18291	0.316	3.909	61	6.70	25	Lys + Ser rich (46%)	−	+	+

**Table 5:** Number of reads and induction coefficient (IC) for putative immune-related genes in *D. virilis* and their homologues in *D. melanogaster. *

*D. virilis *				*D. melanogaster *			
Putative gene	No. of reads		IC	Homologue	No. of reads		IC
	Infected	Naïve			Infected	Naïve	
PG00034	17*	37	0.477	IM14	68**	19	3.162
PG01778	7*	0	infinity	Rho1	16*	7	2.020
PG02420	2*	10	0.208	—	—	—	—

*, **Significant difference from the number of reads for naïve larvae (*P < 0.05, **P < 0.01).
