# Supplementary material for: Extensive Differences in Antifungal Immune Response in Two Drosophila Species Revealed by Comparative Transcriptome Analysis
Source: Int J Genomics. 2013 Sep 10;2013:542139. doi: 10.1155/2013/542139 (PMC3782847; doi:10.1155/2013/542139)

Supplementary Table 1: Number of reads, trimmed mean of M value (TMM) and induction coefficient (IC) for endogenous control genes in *D. virilis* and *D. melanogaster*.

Organism	Symbol	Gene	Infected		Naïve		IC
			No. of reads	TMM	No. of reads	TMM	
D. virilis	RpL32	RpL32	38	1.156	31	0.884	1.307
	Gapdh	GJ20812	0	0	2	0.022	0
		GJ20492	27	0.333	36	0.416	0.800
D. melanogaster	RpL32	RpL32	71	2.216	87	3.073	0.721
	Gapdh	Gapdh1	27	0.351	40	0.589	0.597
		Gapdh2	11	0.134	21	0.289	0.463

Supplementary Table 2: Number of reads, trimmed mean of M value (TMM) and induction coefficient (IC) for recognition, signaling and effector class immune-related genes observed in *D. virilis*.

D. virilis gene	*D. melanogaster* homologue	Infected		Naïve		IC	Functional Class	Notes
		No of reads	TMM	No of reads	TMM			
GJ20666	CG13422	6 *	0.153	0	0	Infinity	Recognition	Beta-glucan binding domain
GJ18161	nimB5	5	0.070	0	0	Infinity	Recognition	Nimrod-related
GJ22101	Corin	1	0.003	0	0	Infinity	Recognition	Scavenger receptor
GJ11092	modSP	1	0.009	0	0	Infinity	Recognition	Scavenger receptor
GJ12160	PGRP-SB1	11 *	0.235	2	0.040	5.864	Recognition	PGRP domain
GJ20388	Mcr	4	0.009	1	0.002	4.265	Recognition	Tep
GJ13216	modSP	10	0.073	5	0.034	2.132	Recognition	Scavenger receptor
GJ19730	crq	2	0.017	1	0.008	2.132	Recognition	Scavenger receptor
GJ15950	PGRP-SA	10	0.208	7	0.137	1.523	Recognition	PGRP domain
GJ16225	TepII	28	0.082	26	0.071	1.148	Recognition	Tep
GJ13386	PGRP-LF	2	0.022	2	0.021	1.066	Recognition	PGRP domain
GJ13383	PGRP-LC	7	0.054	8	0.058	0.933	Recognition	PGRP domain
GJ18075	nimB2	29	0.298	36	0.347	0.859	Recognition	Nimrod-related
GJ18565	PGRP-LE	3	0.034	4	0.042	0.800	Recognition	PGRP domain
GJ18162	nimC2	11	0.063	15	0.081	0.78	Recognition	Nimrod-related
GJ17482	pes	2	0.015	3	0.021	0.71	Recognition	Scavenger receptor
GJ14102	GNBP2	5	0.149	8	0.223	0.666	Recognition	Beta-glucan binding domain
GJ13082	GNBP3	6	0.050	10	0.078	0.640	Recognition	Beta-glucan binding domain
GJ23926	modSP	4	0.026	7	0.042	0.609	Recognition	Scavenger receptor
GJ18229	TepIV	29	0.081	54	0.141	0.573	Recognition	Tep
GJ21683	CG30148	7	0.230	14	0.431	0.533	Recognition	Beta-glucan binding domain
GJ18073	nimB4	1	0.009	3	0.025	0.355	Recognition	Nimrod-related

Supplementary Table 2 continued

GJ18074	nimB3	2 *	0.067	12	0.376	0.178	Recognition	Nimrod-related
GJ11132	r2d2	3	0.039	1	0.012	3.198	Other	.
GJ20897	Dcr-2	4	0.010	5	0.011	0.853	Other	.
GJ15620	et	5	0.028	0	0	Infinity	Signaling	.
GJ18697	Ulp1	5	0.023	0	0	Infinity	Signaling	Protein modification
GJ21884	bsk	2	0.022	0	0	Infinity	Signaling	Kinase
GJ16598	phl	2	0.011	0	0	Infinity	Signaling	.
GJ20084	casp	1	0.034	0	0	Infinity	Signaling	Negative regulator of imd
GJ21215	grass	1	0.027	0	0	Infinity	Signaling	Protease
GJ21265	her	1	0.012	0	0	Infinity	Signaling	Transcription factor
GJ16702	Dredd	1	0.008	0	0	Infinity	Signaling	Protease
GJ16121	Traf4	1	0.008	0	0	Infinity	Signaling	.
GJ20901	Iap2	1	0.008	0	0	Infinity	Signaling	.
GJ14961	Su(var)2-10	1	0.007	0	0	Infinity	Signaling	.
GJ24520	ea	1	0.005	0	0	Infinity	Signaling	Protease
GJ23176	Ser	1	0.003	0	0	Infinity	Signaling	Ligand
GJ12373	msn	9 *	0.024	1	0.002	9.595	Signaling	Kinase
GJ20603	Pvr	15 **	0.038	2	0.005	7.996	Signaling	Receptor
GJ23773	Hel89B	5	0.011	1	0.002	5.331	Signaling	.
tub	tub	7	0.055	2	0.015	3.732	Signaling	.
GJ20044	dom	10	0.013	3	0.004	3.554	Signaling	Transcription factor
Ras1	Ras85D	3	0.065	1	0.020	3.198	Signaling	.
GJ20758	Nup214	3	0.007	1	0.002	3.198	Signaling	Nuclear transport
GJ16165	Pvf2	5	0.055	2	0.020	2.665	Signaling	Ligand
GJ21092	Myd88	5	0.044	2	0.017	2.665	Signaling	.

Supplementary Table 2 continued

GJ18227	ref(2)P	18	0.113	9	0.053	2.132	Signaling	.
GJ11038	Mkk4	4	0.038	2	0.018	2.132	Signaling	Kinase
GJ18559	smt3	2	0.080	1	0.038	2.132	Signaling	Protein modification
GJ10528	mask	16	0.016	9	0.008	1.895	Signaling	.
GJ12202	Uev1A	7	0.197	4	0.105	1.866	Signaling	Protein modification
GJ19711	lwr	5	0.128	3	0.072	1.777	Signaling	Protein modification
GJ24191	Stat92E	13	0.069	8	0.040	1.733	Signaling	Transcription factor
GJ17162	cact	8	0.059	5	0.035	1.706	Signaling	.
GJ19035	ben	3	0.081	2	0.051	1.599	Signaling	Protein modification
GJ16904	hep	4	0.012	3	0.009	1.422	Signaling	Kinase
GJ23481	Rel	7	0.030	6	0.024	1.244	Signaling	Transcription factor
GJ22783	SPE	7	0.071	7	0.067	1.066	Signaling	Protease
GJ10450	cher	6	0.024	6	0.022	1.066	Signaling	.
GJ16233	Dif	4	0.027	4	0.025	1.066	Signaling	Transcription factor
GJ10642	Kay	3	0.016	3	0.015	1.066	Signaling	Transcription factor
GJ21432	POSH	3	0.014	3	0.013	1.066	Signaling	.
GJ22384	Jra	2	0.028	2	0.026	1.066	Signaling	Transcription factor
GJ13209	Rac1	1	0.021	1	0.020	1.066	Signaling	.
GJ12360	Rac2	1	0.021	1	0.020	1.066	Signaling	.
GJ21078	key	1	0.010	1	0.010	1.066	Signaling	Kinase
GJ20083	casp	1	0.008	1	0.007	1.066	Signaling	Negative regulator of imd
GJ10286	Socs36E	1	0.006	1	0.006	1.066	Signaling	Negative regulator of JAKSTAT
GJ24058	mbo	1	0.006	1	0.005	1.066	Signaling	Nuclear transport
GJ15619	dome	1	0.003	1	0.003	1.066	Signaling	Receptor

Supplementary Table 2 continued

GJ15330	hop	1	0.003	1	0.003	1.066	Signaling	Kinase
GJ20945	18w	1	0.003	1	0.003	1.066	Signaling	Toll-like
GJ10951	Sp7	35	0.371	40	0.398	0.933	Signaling	Phenoloxidase cascade
GJ20054	Egfr	5	0.014	6	0.016	0.888	Signaling	Receptor
GJ14938	nec	16	0.126	20	0.148	0.853	Signaling	Protease
GJ18893	Pvf1	3	0.038	4	0.047	0.800	Signaling	Ligand
GJ21412	Spn27A	5	0.046	7	0.061	0.762	Signaling	Phenoloxidase cascade
GJ20786	Stam	7	0.040	10	0.053	0.746	Signaling	.
GJ18599	Ntf-2	2	0.063	3	0.088	0.711	Signaling	Nuclear transport
GJ14460	MP1	14	0.145	22	0.213	0.678	Signaling	Phenoloxidase cascade
GJ23376	srp	10	0.033	16	0.050	0.666	Signaling	Transcription factor
GJ10449	cher	6	0.017	10	0.027	0.640	Signaling	.
GJ15197	psh	14	0.145	25	0.242	0.597	Signaling	Protease
GJ11780	brm	5	0.012	9	0.021	0.592	Signaling	Transcription factor
GJ23435	ben	1	0.027	2	0.051	0.533	Signaling	Protein modification
GJ18753	slpr	1	0.003	2	0.006	0.533	Signaling	.
GJ17781	ush	4	0.011	10	0.026	0.426	Signaling	Transcription factor
GJ21441	smt3	1	0.045	3	0.127	0.355	Signaling	Protein modification
GJ18140	p38b	1	0.011	3	0.032	0.355	Signaling	Kinase
aop	aop	1	0.005	3	0.015	0.355	Signaling	Transcription factor
GJ11480	BG4	1	0.018	4	0.067	0.267	Signaling	.
GJ19441	SPE	3 *	0.033	15	0.155	0.213	Signaling	Protease
GJ22479	Def	53 **	2.445	0	0	Infinity	Effector	Antimicrobial peptide
GJ21173	AttC	47 **	0.818	0	0	Infinity	Effector	Antimicrobial peptide

Supplementary Table 2 continued

Cec2B	CecA1 / CecA2	25 **	1.604	0	0	Infinity	Effector	Antimicrobial peptide
Cec3	CecC	23 **	1.475	0	0	Infinity	Effector	Antimicrobial peptide
GJ22469	Mtk	9 **	0.660	0	0	Infinity	Effector	Antimicrobial peptide
GJ22662	AttD	5	0.113	0	0	Infinity	Effector	Antimicrobial peptide
GJ13808	Tsf2	3	0.015	0	0	Infinity	Effector	Iron binding
GJ15754	Jafrac1	2	0.042	0	0	Infinity	Effector	Gut protection
Ddc	Ddc	2	0.016	0	0	Infinity	Effector	Melanin synthesis cascade
GJ21181	PO45	2	0.012	0	0	Infinity	Effector	Phenoloxidase
Cec1	CecC	1	0.064	0	0	Infinity	Effector	Antimicrobial peptide
GJ12891	Jafrac2	1	0.017	0	0	Infinity	Effector	Gut protection
GJ14144	Duox	1	0.003	0	0	Infinity	Effector	Gut protection
GJ19916	Dpt	104 **	3.812	4	0.138	27.720	Effector	Antimicrobial peptide
GJ19917	DptB	39 **	1.120	3	0.081	13.860	Effector	Antimicrobial peptide
GJ12507	LysD	5	0.146	1	0.027	5.331	Effector	Lysozyme, c-type
GJ20571	AttA	5	0.087	1	0.016	5.331	Effector	Antimicrobial peptide
GJ20572	AttA	49 *	0.856	24	0.393	2.177	Effector	Antimicrobial peptide
GJ20545	Pu	83	1.257	58	0.824	1.526	Effector	Melanin synthesis cascade
GJ10563	Irc	19	0.110	14	0.076	1.447	Effector	Gut protection
GJ11603	Catsup	6	0.056	6	0.053	1.066	Effector	Melanin synthesis cascade
GJ20669	Tsf3	1	0.006	1	0.005	1.066	Effector	Iron binding
GJ21009	CG6426	7	0.177	8	0.190	0.933	Effector	Lysozyme, I-type
GJ15168	Tig	47	0.087	55	0.095	0.911	Effector	Coagulation
GJ10805	yellow-f2	2	0.018	3	0.025	0.711	Effector	Melanin synthesis cascade
GJ15366	Tsf1	111	0.713	172	1.036	0.688	Effector	Iron binding
GJ17981	fon	217 *	1.641	370	2.624	0.625	Effector	Coagulation
GJ21309	IM10	22	0.257	37	0.405	0.634	Effector	IM

Supplementary Table 2 continued

GJ18607	IM4	79 *	7.542	151	13.521	0.558	Effector	IM
GJ21008	CG6426	2	0.051	4	0.095	0.533	Effector	Lysozyme, I-type
GJ21308	IM10	23 *	0.350	51	0.727	0.481	Effector	IM
GJ18065	CG15293	9	0.069	22	0.159	0.436	Effector	Coagulation
GJ13134	CG14823	2	0.036	5	0.085	0.426	Effector	Lysozyme, I-type; destabilase
GJ19885	IM1	37 **	3.302	123	10.296	0.321	Effector	IM
GJ18809	CG16799	1	0.025	5	0.117	0.213	Effector	Lysozyme, c-type
GJ22454	IM23	1	0.039	6	0.218	0.178	Effector	IM
GJ12894	LysD	1	0.029	7	0.187	0.152	Effector	Lysozyme, c-type

Genes are shown in order of induction coefficient at each functional class.

*, ** Significant difference from the number of reads for naïve larvae (* $P < 0.05$, ** $P < 0.01$).

Supplementary Table 3: Number of reads, trimmed mean of M value (TMM) and induction coefficient (IC) for recognition, signaling and effector class immune-related genes observed in *D. melanogaster*.

D. melanogaster gene	Infected		Naïve		IC	Functional Class	Notes
	No. of reads	TMM	No. of reads	TMM			
PGRP-SB1	29 **	0.779	0	0	Infinity	Recognition	PGRP domain
PGRP-SC1b	11 **	0.288	0	0	Infinity	Recognition	Amidase degradation
PGRP-SB2	9 **	0.225	0	0	Infinity	Recognition	PGRP domain
Mcr	4 *	0.011	0	0	Infinity	Recognition	Tep
GNBP2	3	0.035	0	0	Infinity	Recognition	Beta-glucan binding domain
Sr-CIV	2	0.024	0	0	Infinity	Recognition	Scavenger receptor
nimB1	1	0.013	0	0	Infinity	Recognition	Nimrod-related
PGRP-SC1a	1	0.030	0	0	Infinity	Recognition	Amidase degradation
PGRP-SC2	20 **	0.603	3	0.102	5.891	Recognition	Amidase degradation
TepII	188 **	0.708	31	0.132	5.359	Recognition	Tep
nimC2	43 **	0.310	9	0.073	4.222	Recognition	Nimrod-related
GNBP3	15 **	0.164	4	0.049	3.313	Recognition	Beta-glucan binding domain
CG13422	17 **	0.569	5	0.189	3.004	Recognition	Beta-glucan binding domain
TepIV	37 **	0.131	13	0.052	2.515	Recognition	Tep
crq	5	0.043	2	0.020	2.209	Recognition	Scavenger receptor
PGRP-SA	7	0.103	3	0.050	2.062	Recognition	PGRP domain
TepI	7	0.028	3	0.013	2.062	Recognition	Tep
PGRP-SD	27 **	0.626	13	0.341	1.835	Recognition	PGRP domain
modSP	2	0.014	2	0.016	0.884	Recognition	Scavenger receptor
PGRP-LC	1	0.009	1	0.010	0.884	Recognition	PGRP domain
PGRP-LE	1	0.009	1	0.010	0.884	Recognition	PGRP domain
pes	1	0.007	1	0.007	0.884	Recognition	Scavenger receptor
PGRP-LB	1	0.013	1	0.015	0.884	Recognition	Amidase degradation
nimB2	24	0.279	29	0.382	0.731	Recognition	Nimrod-related
nimB4	4	0.045	5	0.064	0.707	Recognition	Nimrod-related
nimB3	24	0.898	44	1.864	0.482	Recognition	Nimrod-related
CG30148	1	0.026	2	0.058	0.442	Recognition	Beta-glucan binding domain
emp	1	0.007	3	0.025	0.295	Recognition	Scavenger receptor
Dcr-2	3	0.009	2	0.007	1.325	Other	.
Rel	14 **	0.067	0	0	Infinity	Signaling	Transcription factor
aop	6 **	0.026	0	0	Infinity	Signaling	Transcription factor
brm	5 *	0.016	0	0	Infinity	Signaling	Transcription factor

Supplementary Table 3 continued

Myd88	4 *	0.019	0	0	Infinity	Signaling	.
ush	3	0.008	0	0	Infinity	Signaling	Transcription factor
Mpk2	3	0.034	0	0	Infinity	Signaling	Kinase
ird5	3	0.020	0	0	Infinity	Signaling	Kinase
slpr	3	0.010	0	0	Infinity	Signaling	.
lwr	3	0.045	0	0	Infinity	Signaling	Protein modification
hop	3	0.010	0	0	Infinity	Signaling	Kinase
Ulp1	3	0.010	0	0	Infinity	Signaling	Protein modification
tamo	2	0.009	0	0	Infinity	Signaling	Nuclear transport
Traf4	2	0.015	0	0	Infinity	Signaling	.
Pvf2	1	0.008	0	0	Infinity	Signaling	Ligand
edl	1	0.011	0	0	Infinity	Signaling	.
Dsor1	1	0.009	0	0	Infinity	Signaling	Kinase
mbo	1	0.007	0	0	Infinity	Signaling	Nuclear transport
Rac2	1	0.011	0	0	Infinity	Signaling	.
CG6361	15 **	0.185	1	0.014	13.254	Signaling	Protease
cact	11 **	0.081	1	0.008	9.720	Signaling	.
dom	8 *	0.085	1	0.012	7.069	Signaling	Transcription factor
Rac1	4	0.037	1	0.010	3.534	Signaling	.
spz	4	0.033	1	0.010	3.534	Signaling	Ligand
Stat92E	11 *	0.050	3	0.016	3.240	Signaling	Transcription factor
srp	18 **	0.080	5	0.025	3.181	Signaling	Transcription factor
phl	32 **	0.135	9	0.043	3.142	Signaling	.
mask	10 *	0.012	3	0.004	2.945	Signaling	.
spirit	22 **	0.231	7	0.083	2.777	Signaling	Protease
hep	6	0.031	2	0.012	2.651	Signaling	Kinase
emb	6	0.022	2	0.008	2.651	Signaling	Nuclear transport
Pvr	8	0.026	3	0.011	2.356	Signaling	Receptor
Sp7	13	0.112	6	0.059	1.914	Signaling	Phenoloxidase cascade
ben	15	0.138	7	0.073	1.893	Signaling	Protein modification
Egfr	6	0.022	3	0.012	1.767	Signaling	Receptor
Stam	4	0.023	2	0.013	1.767	Signaling	.
Ntf-2	4	0.022	2	0.012	1.767	Signaling	Nuclear transport
Hel89B	2	0.005	1	0.003	1.767	Signaling	.
key	2	0.025	1	0.014	1.767	Signaling	Kinase
Mkk4	2	0.013	1	0.008	1.767	Signaling	Kinase
Dif	2	0.013	1	0.007	1.767	Signaling	Transcription factor

Supplementary Table 3 continued

tub	2	0.017	1	0.009	1.767	Signaling	.
imd	2	0.025	1	0.014	1.767	Signaling	.
dome	3	0.010	2	0.008	1.325	Signaling	Receptor
grass	3	0.038	2	0.029	1.325	Signaling	Protease
nec	18	0.186	14	0.164	1.136	Signaling	Protease
ref(2)P	10	0.075	8	0.068	1.104	Signaling	.
SPE	7	0.093	6	0.090	1.031	Signaling	Protease
MP1	9	0.101	9	0.114	0.884	Signaling	Phenoloxidase cascade
Jra	3	0.033	3	0.037	0.884	Signaling	Transcription factor
msn	2	0.006	2	0.007	0.884	Signaling	Kinase
Iap2	1	0.008	1	0.009	0.884	Signaling	.
Dredd	1	0.008	1	0.0010	0.884	Signaling	Protease
ytr	1	0.008	1	0.009	0.884	Signaling	.
Traf-like	1	0.007	1	0.008	0.884	Signaling	.
kay	1	0.004	1	0.004	0.884	Signaling	Transcription factor
pnt	1	0.006	1	0.007	0.884	Signaling	Transcription factor
Uev1A	7	0.087	9	0.127	0.687	Signaling	Protein modification
psh	3	0.030	4	0.046	0.663	Signaling	Protease
Spn27A	2	0.015	3	0.025	0.589	Signaling	Phenoloxidase cascade
Su(var)2-10	2	0.016	3	0.026	0.589	Signaling	.
cher	4	0.019	7	0.038	0.505	Signaling	.
smt3	3	0.068	9	0.229	0.295	Signaling	Protein modification
CecC	35 **	1.521	0	0	Infinity	Effector	Antimicrobial peptide
CecA1	14 **	0.663	0	0	Infinity	Effector	Antimicrobial peptide
Def	11 **	0.461	0	0	Infinity	Effector	Antimicrobial peptide
CecB	7 **	0.288	0	0	Infinity	Effector	Antimicrobial peptide
dro5	6 **	0.276	0	0	Infinity	Effector	Antimicrobial peptide
Hml	2	0.003	0	0	Infinity	Effector	Coagulation
CG18107	2	0.118	0	0	Infinity	Effector	IM
TotB	2	0.063	0	0	Infinity	Effector	Tot
CecA2	2	0.094	0	0	Infinity	Effector	Antimicrobial peptide
Tsf3	1	0.007	0	0	Infinity	Effector	Iron binding
AttC	252 **	4.684	2	0.042	111.333	Effector	Antimicrobial peptide
Dpt	343 **	11.568	24	0.916	12.628	Effector	Antimicrobial peptide
DptB	80 **	2.974	6	0.252	11.781	Effector	Antimicrobial peptide
Pu	79 **	0.687	7	0.069	9.972	Effector	Melanin synthesis cascade

Supplementary Table 3 continued

TotC	10 **	0.311	1	0.035	8.836	Effector	Tot
IM18	62 **	1.403	8	0.205	6.848	Effector	IM
Mtk	380 **	23.719	52	3.673	6.457	Effector	Antimicrobial peptide
Dro	192 **	4.237	27	0.674	6.283	Effector	Antimicrobial peptide
Jafrac1	5	0.083	1	0.019	4.418	Effector	Gut protection
yellow-f	23 **	0.277	6	0.082	3.387	Effector	Melanin synthesis cascade
IM14	68 **	5.101	19	1.613	3.162	Effector	IM
AttA	96 **	2.113	27	0.673	3.142	Effector	Antimicrobial peptide
IM4	56 **	2.194	16	0.709	3.093	Effector	IM
IM10	355 **	6.147	116	2.273	2.704	Effector	IM
IM1	247 **	11.541	82	4.336	2.662	Effector	IM
AttB	74 **	1.428	27	0.590	2.422	Effector	Antimicrobial peptide
IM2	139 **	6.250	62	3.155	1.981	Effector	IM
Tsf1	145 **	1.209	68	0.642	1.884	Effector	Iron binding
IM23	6	0.216	3	0.122	1.767	Effector	IM
TotA	182 **	5.213	98	3.177	1.641	Effector	Tot
Drs	551 **	23.817	299	14.627	1.628	Effector	Antimicrobial peptide
Tig	22 **	0.053	12	0.033	1.620	Effector	Coagulation
CG15293	9	0.126	5	0.079	1.590	Effector	Coagulation
IM3	330 **	18.401	188	11.864	1.551	Effector	IM
CG33470	9	0.167	6	0.126	1.325	Effector	IM
Irc	26	0.182	22	0.174	1.044	Effector	Gut protection
CG16799	7	0.164	10	0.265	0.619	Effector	Lysozyme, c-type
fon	80	0.696	121	1.192	0.584	Effector	Coagulation
Catsup	2	0.018	8	0.082	0.221	Effector	Melanin synthesis cascade

Genes are shown in order of induction coefficient at each functional class.

*, ** Significant difference from the number of reads for naïve larvae (* $P < 0.05$, ** $P < 0.01$).

Supplementary Table 4: Number of reads, induction coefficient (IC) and predicted function of the putative genes (PG) in *D. virilis*.

Putative gene	No. of reads		IC	Swissprot ID	Definition	Organism	Immune-related
	Infected	Naïve					
PG00034	17	37	0.48	P83869	Immune-induced peptide 14	*Drosophila melanogaster*	+
PG00098	1131	814	1.44	A7Y3K2	Putative membrane protein ycf1	*Ipomoea purpurea*	-
PG00273	32	15	2.22	P05389	60S acidic ribosomal protein P2	*Drosophila melanogaster*	-
PG00326	13	4	3.38	P29742	Clathrin heavy chain	*Drosophila melanogaster*	-
PG00604	11	1	11.44	P60892	Ribose-phosphate pyrophosphokinase 1	*Rattus norvegicus*	-
PG00667	17	5	3.53	P61255	60S ribosomal protein L26	*Mus musculus*	-
PG00683	17	54	0.33	P61210	ADP-ribosylation factor 1	*Locusta migratoria*	-
PG01080	36	86	0.43	O44390	Acyl-CoA Delta(11) desaturase	*Trichoplusia ni*	-
PG01083	12	0	Infinity	P54385	Glutamate dehydrogenase, mitochondrial	*Drosophila melanogaster*	-
PG01215	53	122	0.45	P07701	Salivary glue protein Sgs-5	*Drosophila melanogaster*	-
PG01259	21	7	3.12	P02553	Tubulin alpha chain (Fragment)	*Lytechinus pictus*	-
PG01319	15	0	Infinity	P23194	Uricase	*Drosophila virilis*	-
PG01327	8	1	8.32	Q7KN62	Transitional endoplasmic reticulum ATPase TER94	*Drosophila melanogaster*	-
PG01341	10	0	Infinity	P79398	Eukaryotic translation initiation factor 4 gamma 2	*Oryctolagus cuniculus*	-
PG01370	1	14	0.074	Q03168	Lysosomal aspartic protease	*Aedes aegypti*	-
PG01376	2	11	0.19	Q962Q6	40S ribosomal protein S24	*Spodoptera frugiperda*	-
PG01460	62	27	2.39	P31403	V-type proton ATPase 16 kDa proteolipid subunit	*Manduca sexta*	-
PG01494	17	4	4.42	P20007	Phosphoenolpyruvate carboxykinase [GTP]	*Drosophila melanogaster*	-
PG01518	1	8	0.13	Q8T8R1	CCHC-type zinc finger protein CG3800	*Drosophila melanogaster*	-
PG01778	7	0	Infinity	P48148	Ras-like GTP-binding protein Rho1	*Drosophila melanogaster*	+
PG01865	55	105	0.54	P54361	Ornithine decarboxylase antizyme	*Drosophila melanogaster*	-
PG01979	18	39	0.48	P02707	Hepatic lectin	*Gallus gallus*	-
PG01980	15	0	Infinity	P11997	Larval serum protein 1 gamma chain	*Drosophila melanogaster*	-

Supplementary Table 4 continued

PG02420	2	10	0.21	Q5I2E5	Ficolin-2	*Bos taurus*	+
PG02437	10	30	0.35	Q9GPH3	Activating transcription factor of chaperone	*Bombyx mori*	-
PG03038	46	22	2.17	Q9NJH0	Elongation factor 1-gamma	*Drosophila melanogaster*	-
PG03151	6	0	Infinity	P60517	Gamma-aminobutyric acid receptor-associated protein	*Rattus norvegicus*	-

Supplementary Figure 1

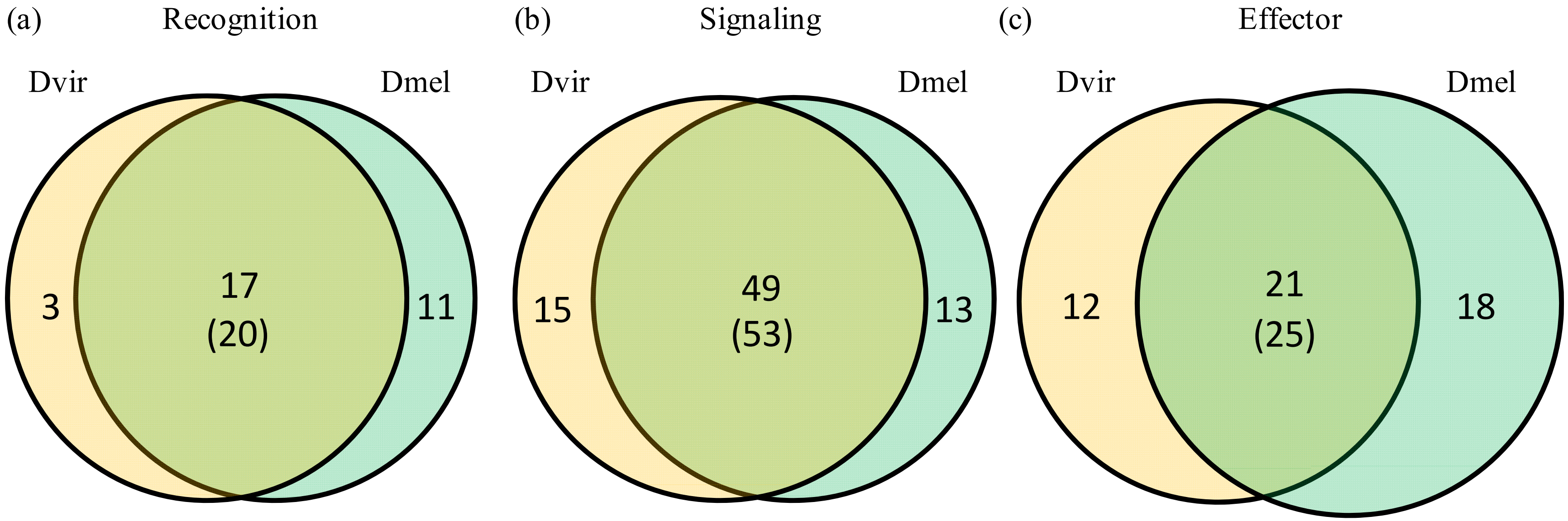

Supplement: Supplementary file 1 — Supplementary Table 1: Number of reads, trimmed mean of M value (TMM) and induction coefficient (IC) for endogenous control genes in D. virilis and D. melanogaster. Supplementary Table 2: Number of reads, trimmed mean of M value (TMM) and induction coefficient (IC) for recognition, signaling and effector class immune-related genes observed in D. virilis. Supplementary Table 3: Number of reads, trimmed mean of M value (TMM) and induction coefficient (IC) for recognition, signaling and effector class immune-related genes observed in D. melanogaster. Supplementary Table 4: Number of reads, induction coefficient (IC) and predicted function of the putative genes (PG) in D. virilis. Supplementary Figure 1: Venn diagrams that represent the numbers of expressed immune-related genes for recognition (a), signaling (b) and effectors (c) observed in the Penicillium-infected D. virilis (Dvir) and D. melanogaster (Dmel) larvae. The numbers in parentheses indicate the numbers of duplicated genes in D. virilis. [file 542139.f1.pdf]
